# Association of organs-crosstalk with the pathogenesis of osteoarthritis: cartilage as a key player

**DOI:** 10.3389/fendo.2025.1593658

**Published:** 2025-06-05

**Authors:** Yingda Qin, Jingkai Di, Zijian Guo, Shuai Chen, Chuan Xiang

**Affiliations:** Second Hospital of Shanxi Medical University, Taiyuan, Shanxi, China

**Keywords:** osteoarthritis, organs-crosstalk, cartilage, chondrocyte, therapeutic strategy

## Abstract

Degeneration of articular cartilage is the hallmark pathologic change in osteoarthritis (OA). Cartilage not only serves as a shock-absorbing structure for movement but is also regulated by organs other than bone, while chondrocytes secrete cytokines that influence these organs. The concept of organ axis refers to the regulatory pathways formed between organs via cytokine signaling. The communication network established between cartilage and other organs constitutes the cartilage-organ axis. Through this axis, these organs regulate articular chondrocyte proliferation and apoptosis. It is evident that chondrocytes play a central role in connecting various organs to the progression of OA, prompting interest in strategies to intervene in cartilage damage by modulating the organ axis. This review presents, for the first time, a system summarizing the effects of the extraosseous system on cartilage through various factors that alter OA progression. The aim is to fully elucidate the effects of different organs on cartilage, thus providing insights into the treatment of OA and systemic diseases.

## 1 Introduction

### 1.1 Pathologic changes of OA

OA is one of the fastest-growing health issues globally (1), with the knee being the most commonly affected joint. Knee osteoarthritis (KOA) is a chronic and disabling condition with multiple etiologies that impacts the entire knee joint (2, 3). The burden of OA continues to rise globally, with studies suggesting that the disease will affect approximately 7.6 per cent of the world’s population by 2024 (4), and KOA ranks as the 11th leading cause of disability globally (5), particularly as the population ages and the prevalence of obesity increases. As a bearing or hinge structure, joints not only provide attachment and support for muscles and ligaments but also facilitate various movements. Traditionally, the primary pathological change in OA has been recognized as the degeneration of articular cartilage. However, a more comprehensive perspective is now emerging, viewing OA as a disease that encompasses the entire joint structure, including cartilage, synovium, subchondral bone, ligaments, and menisci (6). Despite this broader understanding, degeneration of articular cartilage remains the earliest and most characteristic pathological change observed in the progression of OA. In this article, we aim to provide an overview of the pathological changes that occur in cartilage as OA advances. The degeneration of cartilage is the most characteristic pathological change observed in OA (7, 8). When articular cartilage is subjected to mechanical stress, inflammation, metabolic disturbances, immune responses, and genetic factors (9), signaling pathways such as Wnt/β-catenin, NF-κB, MAPK, and PI3K/AKT are abnormally activated in chondrocytes. This ultimately results in mitochondrial dysfunction, which leads to chondrocyte destruction (10). Under normal conditions, cartilage functions as a cushion and reduces joint friction. However, when cartilage degenerates, its surface integrity is compromised, resulting in the release of components such as proteoglycans and collagen from the cartilage matrix, which form ‘wear’ particles (11). These particles are phagocytosed by synovial macrophages, activating synovial cells to release various pro-inflammatory cytokines, including tumor necrosis factor-alpha (TNF-alpha), interleukin-1 (IL-1), and interleukin-6 (IL-6), thereby triggering an inflammatory response in the synovium (12). Consequently, cartilage damage is exacerbated, and its protective effect on the subchondral bone is diminished, exposing the subchondral bone to abnormal mechanical stress (13). This altered mechanical stress can incite microfracture and repair responses in the subchondral bone. During the repair process, the activity of cells such as osteoblasts and osteoclasts increases, leading to the remodeling of the subchondral bone and resulting in changes such as osteophyte formation and osteosclerosis (14–16). In the pathogenesis of arthritis, cartilage degeneration, synovial changes, and subchondral bone alterations are interrelated and synergistic, creating a complex pathophysiologic network that drives disease progression. Cartilage is the first part to degenerate, so we focus on cartilage changes. The proliferation, degeneration, and apoptosis of chondrocytes are regulated by inter-organ crosstalk, indicating that the onset and progression of OA are also influenced by other organs (17–20).

### 1.2 Relationship between organs-crosstalk and OA

Since 2000, with the advancements in systems biology and multi-omics techniques, the term “organ crosstalk” has become increasingly prevalent. Originally, this concept was used to describe the intricate interactions between organs that transmit signaling molecules (e.g., hormones, cytokines, metabolites) via the circulatory system (e.g., blood, lymph) (21–28). Despite the complexity and diversity of inter-organ crosstalk mechanisms, recent years have seen significant progress in elucidating these processes (29, 30). The perspective of inter-organ crosstalk offers novel insights, providing innovative solutions for the treatment of various diseases (31). In recent years, continuous technological innovation has clarified the study of cross-system communication involving cytokines, extracellular vesicles, hormones, and neurotransmitters. This advancement enables scientists to analyze the complex interaction mechanisms between bones, joints, and various organs, including the brain, lungs, liver, heart, and kidneys, in greater depth. Consequently, it promotes the rapid development of research on the bone-organ axis.

**Figure 1:**
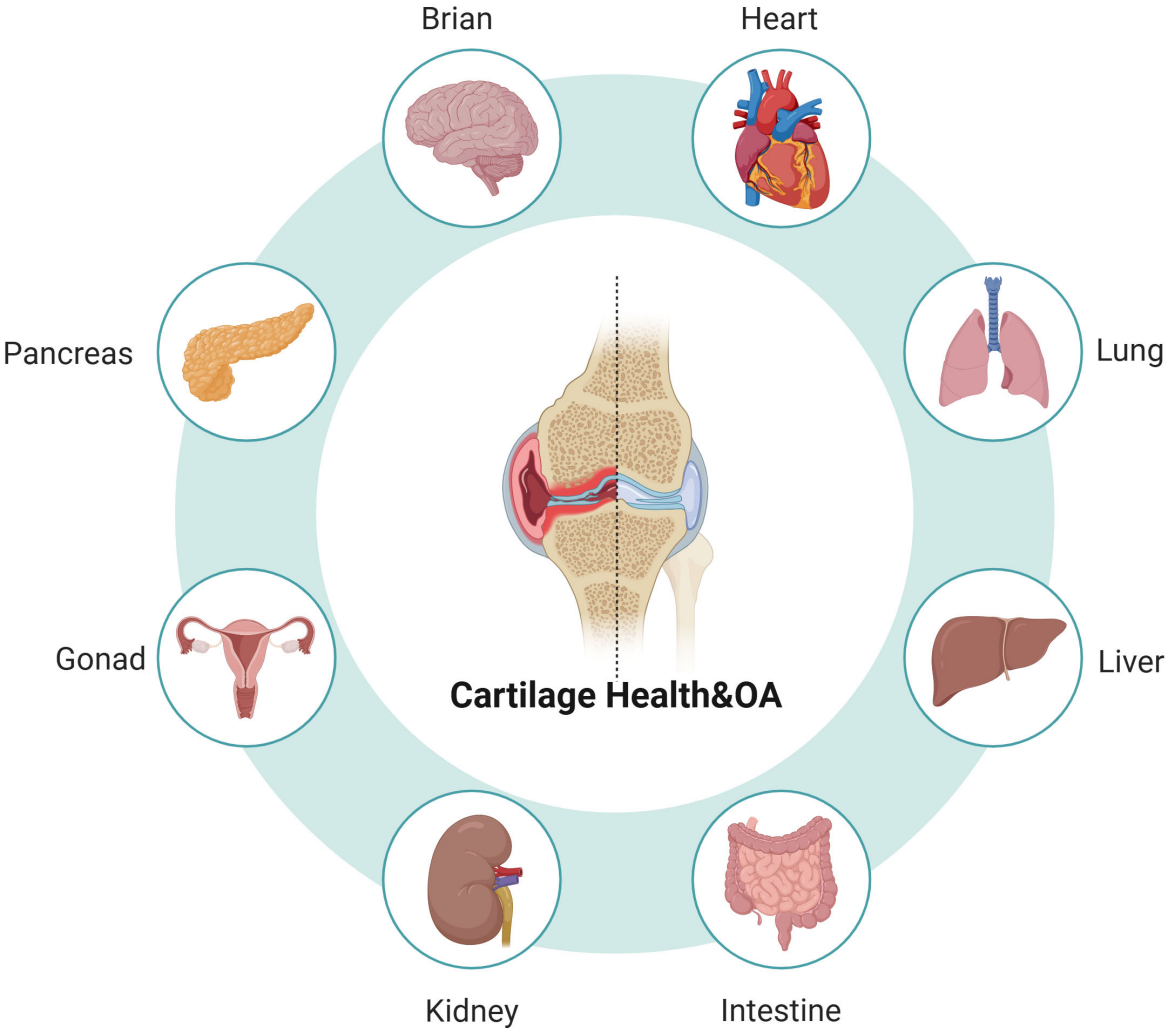
Cartilage is affected by organs throughout the body and also affects some of the organs.

Organ crosstalk alters chondrocyte metabolism by delivering cytokines and metabolites or by influencing macrophage polarization (32). Although numerous mechanisms have been identified, there is a notable lack of comprehensive summaries and systematic analyses in this research area. In recent years, significant interactions between organs such as the heart, brain, liver, and lungs with the osteoarticular system have been identified, profoundly affecting bone and cartilage metabolism and related pathologies (**Tables 1**, **2**). Additionally, growing evidence suggests that organs including the intestines, kidneys, gonads, and pancreas also play a regulatory role in metabolic changes within articular cartilage (**Figure 1**). There is an emerging perspective that fat, muscle, and even bone itself may gradually influence the health of articular cartilage (**Table 3**). Although these tissues are not classified as organs, their ubiquitous presence in the body underscores their importance in research. Joint tissues, as a crucial component of the skeletal system, are also influenced by other internal organs during homeostasis and disease progression, with alterations in articular cartilage being particularly significant. A deeper understanding of the mechanisms underlying bone-organ-axis interactions could facilitate the development of targeted therapies for bone-related and systemic diseases. For instance, by focusing on key molecules or signaling pathways within a specific bone-organ axis, more effective therapeutic drugs and strategies can be devised to enhance treatment efficacy while minimizing adverse effects. In this review, we summarize the relevant mechanisms identified thus far, particularly those related to cartilage damage in OA. By understanding these mechanisms, we aim to provide insights for the prevention and treatment of OA.

**Table 1:** Effects of extraosseous organs on cartilage.

Factor	Organ sources	key function	References
Irisin	Mostly myocardium, also muscle	Increases chondrocyte growth and ECM accumulation.	(38–41)
oxLDL	Cardiovascular system	Impairs mitochondrial autophagy.	(42–46)
Cholesterol		Promotes ECM degradation.	(47, 48)
VEGF	Liver, vascular endothelial cells	Counteracts TNF-α-induced hypertrophy.	(49)
ncRNA-MIR4435-2HG65	Lung tumour	Promotes chondrocyte proliferation and inhibits chondrocyte apoptosis.	(50–52)
CXCR4	Chondrocyte	High chondrocyte expression in COVID-19 patients, leading to cartilage degradation.	(53)
CCN3	Brain	Ameliorates chondrocyte apoptotic effects and attenuates ECM degradation.	(11, 54–60)
MT		Upregulates HA synthesis.	(61)
NGF	Sensory neuron	Promotes acid-induced chondrocyte apoptosis.	(62)
SP		Promotes M2 polarization of macrophages, inhibits chondrocyte apoptosis.	(63–65)
CGRP		Biphasic effects: reduces oxidative stress in healthy state; promotes inflammatory response and extracellular matrix degradation in fine cartilage in pathological state.	(66, 67)
Sema3A		Inhibits cartilage degradation.	(68, 69)
IGF-1	Liver	Inhibits NF-κB signaling and prevents chondrocyte apoptosis by inhibiting ROS production.	(70–73)
GLP	Intestine	Activates GLP-1R to protect chondrocytes from IL-1β or TGs-induced endoplasmic reticulum stress and apoptosis.	(74–78)
SCFAs	Ovary	Inhibits osteoclast activity and modulating the inflammatory response	(79, 80)
CAT		Reduces iron death-dependent chondrocyte injury.	(81)
IAA&IPA		Promotes chondrocyte proliferation.	(82)
Estrogen (ERα+)		Activation of ERα attenuates chondrocyte damage.	(83–85)
Estrogen (GPER+)	pancreas	Activates GPER and decrease intrachondrocyte ROS levels.	(86)
Insulin		low-dose cartilage protection/high-dose cartilage damage.	(87–94)
EC-Exos	Vascular endothelial cell	Inhibits chondrocyte autophagy, induces apoptosis.	(95)

**Table 2:** Effects of cartilage on extraosseous organs.

Factor	Sources	Target cells	Effect	References
miR-214-3p	OA Chondrocyte	Vascular endothelial cell	Inhibits endothelial cell migration and angiogenesis.	(97, 98)
lactic acid	Chondrocyte	Vascular endothelial cell	Promotes angiogenesis.	(99, 100)
YKL-40		Bronchial epithelial cell	Involved in airway inflammation and airway remodeling processes.	(101–103)
CS		Interstitial lung, hepatocytes	Deposits in the interstitium of the lungs and liver and induces pulmonary fibrosis.	(104–106)
FGF-23	Bone	kidney	Increases expression of ColX, Mmp13 and Mmp9 in chondrocytes.	(107)

**Table 3:** Effects of extraosseous tissue on cartilage.

Factor	Organ sources	key function	References
Lipocalin	Adipose tissue	Initiates autophagy, thereby protecting chondrocytes and promoting macrophage clearance of apoptotic chondrocytes.	(111)
FFA		Upregulates Nupr1 expression causing chondrocyte damage.	(112)
Leptin		Elevates ROS levels, and promotes cartilage apoptosis.	(113–116)
MSCIPFP- Exos	Adipose mesenchymal stem cells	Inhibits mTOR, inhibits chondrocyte apoptosis by carrying miR-100-5p into chondrocytes.	(117)
FNIP1	Muscle	Promotes post-traumatic cartilage formation.	(118, 119)
TGF-β	Bone	Protects cartilage at normal concentrations, promotes cartilage degeneration at abnormally high concentrations.	(120, 121)

## 2 Cross-talk between cartilage and other organs

### 2.1 Brain and peripheral nerve-cartilage axis

The influence of the nervous system on cartilage is paramount among all biological systems. The concept of the brain-bone axis has been proposed (33), highlighting the integral roles of both the central and peripheral nervous systems (34–37). Notably, alterations in circadian rhythms can also impact cartilage health. This section summarizes the effects of central and peripheral nerves on chondrocyte proliferation and apoptosis.

#### 2.1.1 Brain and peripheral nerve to cartilage

The brain plays a vital role in regulating cartilage metabolism. This section describes the effects of cellular communication network factor 3 (CCN3), melatonin (MT), and parathyroid hormone (PTH), all of which are produced by the central nervous system and regulated by circadian rhythms, on cartilage. Study finds correlation between OA degenerative changes and CCN3 expression (11). CCN3, a member of the CCN (Cyr61, Ctgf, NOV) family, has been shown to regulate various inflammatory responses (54). Recent findings indicate that CCN3 ameliorates the apoptotic effects on chondrocytes through the activation of the PI3K/AKT/mTOR pathway and enhances the degradation of cartilage matrices (55), including collagen type II and aggregated proteoglycans (56). Furthermore, CCN3 has been identified as being secreted by KISS1 neurons in the arcuate nucleus (ARC KISS1), which increases the potential for bone and cartilage formation, thereby improving cartilage quality (57). The central nervous system is implicated in the regulation of circadian rhythms and has been shown to influence the hypothalamic-pituitary-adrenal axis and hormones, thereby impacting the progression of OA (58–60). Changes in serum concentrations of PTH, regulated by the central nervous system, exhibit diurnal variations, suggesting that cartilage proliferation is also influenced by daily activity rhythms (96). MT, a hormone secreted by the pineal gland, is regulated by circadian rhythms. It has been demonstrated that MT protects chondrocytes by upregulating hyaluronic acid synthesis, inhibiting the NF-kB signaling pathway, and repairing the redox environment and membrane potential within mitochondria (61).

Peripheral nerves not only mediate the production of nociception but also secrete factors that influence cartilage health, including substance P (SP), calcitonin gene-related peptide (CGRP), and semaphorin 3A (Sema3A), which regulate cartilage metabolism. Nerve Growth Factor (NGF) plays a critical role in the development of OA pain by stimulating sensory neurons to produce nociception, which is the primary source of pain in OA (62). Blocking NGF has been shown to reduce pain sensitivity in OA patients (108). Certain factors, such as SP and CGRP, are involved in both nociception production and cartilage metabolism (109, 110). They serve a dual purpose: transducing nociception and regulating chondrocyte metabolism. SP, a peptide primarily secreted by neurons (63), stimulates the secretion of pro-inflammatory chemokines by macrophages and mast cells (64). Additionally, in the context of tissue injury, SP promotes M2 polarization of macrophages, which mitigates inflammatory responses and inhibits chondrocyte apoptosis in OA (65). CGRP exhibits biphasic regulation of chondrocytes: in normal cartilage, it may protect chondrocytes from apoptosis by inhibiting the release of inflammatory factors (e.g., IL-1β, TNF-α), reducing oxidative stress, and counteracting the apoptotic effects of NGF by preventing the abnormal growth of peripheral sensory nerves into cartilage tissue (66). However, CGRP expression is elevated in OA cartilage, where it promotes inflammatory responses and degradation of the cartilage extracellular matrix, with excessive release in degenerating tissues potentially exacerbating chronic pain (67, 110). Sema3A, another protein secreted by sensory neurons (68), maintains cartilage homeostasis and inhibits nerve growth into cartilage during OA. It exerts a protective effect against cartilage degradation through the activation of the PI3K signaling pathway (69).

Neurodegenerative diseases, particularly Alzheimer’s disease (AD), have a significant interplay with OA, with evidence suggesting that AD may promote the development of OA. The pathogenesis of OA is closely associated with AD (33), as indicated by recent studies revealing that patients with KOA are at an elevated risk of developing AD-related dementia (ADRD) (122, 123). Current understanding of the mechanisms underlying the interaction between AD and OA highlights a notable increase in serum IL-6 levels in osteoarthritis, which subsequently leads to heightened levels of inflammatory cytokines in peripheral blood, contributing to brain inflammation (124).

#### 2.1.2 Cartilage to brain

The mechanisms by which healthy chondrocytes influence the nervous system remain largely unexplored; however, cartilage in the context of OA significantly contributes to various neurological disorders. Degenerated chondrocytes associated with OA are implicated in the emergence of lesions characteristic of AD. During the progression of OA, peripheral inflammation persists, marked by elevated levels of inflammatory mediators such as IL-1β, IL-6, and TNF. Notably, these cytokines, along with IL-18, have the capacity to cross the blood-brain barrier (BBB) (125), potentially inducing brain lesions. Moreover, inflammatory cytokines resulting from chondrocyte damage may expedite the deposition of amyloid beta (Aβ) and the advancement of neuroinflammation (126–128). This process leads to the hyperphosphorylation of tau proteins and subsequent neuronal loss. The intracellular aggregation of abnormally phosphorylated tau proteins results in the formation of neurofibrillary tangles (NFTs), ultimately culminating in neuronal degeneration, which, when sufficiently severe, can lead to the development of Alzheimer’s disease (129).

While the potential treatment avenues mentioned above show promise for continued research, several challenges persist. These include the necessity for a more profound understanding of the intricate interactions between the nervous system and osteoarthritis (OA). Given the dynamic nature of brain and peripheral nerve functions, along with the significant individual differences that must be considered, a personalized medicine approach may be essential to optimize treatment outcomes. In other words, targeting cartilage improvement by modulating nervous system function for OA treatment represents an emerging field that may provide new opportunities for integrated brain-joint therapy. Nevertheless, further research is required to fully elucidate the mechanisms of action and to develop targeted and effective treatments.

### 2.2 Heart-cartilage axis

The correlation between cardiovascular disease (CVD) and OA has been well established, with numerous studies demonstrating that the comorbidities of either condition are directly and significantly associated with an increased risk of developing the other (130). Both conditions often exhibit common pathological mechanisms in their pathogenesis, such as oxidative stress, local microvascular remodeling, and hyperlipidemia. Additionally, the daily behavioral patterns of OA patients contribute to an increased incidence of CVD (131, 132). Similarly, reduced cardiac function can promote the progression of OA (133). There are also molecular mechanisms that interact between the two conditions (134–136), highlighting the importance of exploring the heart-cartilage axis as a potential avenue for new therapeutic options for OA.

#### 2.2.1 Cardiovascular to cartilage

The cardiovascular system produces specific cytokines and vesicles that are modified and released into the bloodstream to influence chondrocytes. Cardiomyocytes exhibit high expression levels of a protein known as fibronectin type III domain-containing protein 5 (FNDC5) (38). FNDC5 is a transmembrane protein composed of an extracellular irisin structural domain and an intracytoplasmic C-terminal structural domain. Irisin, a soluble peptide cleaved from the extracellular structural domain of FNDC5 (39), has been shown to promote chondrocyte growth and extracellular matrix (ECM) accumulation by enhancing mitochondrial autophagy in chondrocytes (40, 41). Vascular endothelial growth factor (VEGF), primarily produced by vascular endothelial cells and the liver, has been implicated in exacerbating the progression of OA. Inhibition of VEGF expression not only enhances chondrogenic differentiation but also protects chondrocytes from tumor necrosis factor-alpha (TNF-α)-induced hypertrophic changes (49). Furthermore, exosomes derived from vascular endothelial cells (EC-Exos) act as negative regulators of chondrocytes, inhibiting chondrocyte autophagy. The entry of EC-Exos into chondrocytes elevates cellular reactive oxygen species (ROS) levels and induces apoptosis by compromising chondrocyte resistance to oxidative stress (95). Factors produced by cardiomyocytes or vascular endothelial cells are transported to the joints via the bloodstream. Collectively, the effects of FNDC5 and VEGF are well-established (137); however, the impact of exosomes produced by the cardiovascular system on chondrocytes remains unclear. Additionally, the influence of exosomes secreted by the cardiovascular system in various states on the joints has yet to be conclusively determined.

Metabolites associated with CVD)are significant factors influencing chondrocyte metabolism, particularly those resulting from abnormal lipid metabolism, which exert the most profound effects. CVD is characterized by elevated blood levels of low-density lipoprotein (LDL) and cholesterol. Excess LDL undergoes oxidation, resulting in oxidized LDL (oxLDL). Both oxLDL and cholesterol contribute to processes such as synovial inflammation, cartilage degeneration, and bone deformation (42–45). Nakagawa et al. demonstrated that oxLDL induces damage to chondrocytes *in vitro (* 138), subsequent studies indicated that oxLDL enhances cartilage degradation by increasing the expression of monocyte chemoattractant protein 1(MCP-1) in chondrocytes (46). Furthermore, oxLDL impairs autophagic flux, leading to autophagic dysfunction and promoting chondrocyte apoptosis by elevating p62/SQSTM1 levels and inhibiting the activity of transcription factor EB (TFEB) (139). Elevated cholesterol levels significantly enhance synovial activation and heterotopic bone formation in early collagenase-induced OA (140, 141). In chondrocytes, cholesterol and its oxidative metabolites directly activate retinoic acid-related orphan receptor α (RORα), contributing to OA by upregulating matrix-degrading enzymes (142). Additionally, increased blood cholesterol levels, and consequently higher cholesterol concentrations in joint fluid, downregulate low-density lipoprotein receptor-related protein 3(LRP3) expression in chondrocytes while upregulating syndecan-4 through the activation of the Ras signaling pathway, thereby inducing degeneration of the cartilaginous ECM (47, 48). Disturbances in lipid metabolism within the cardiovascular system can lead to increased oxidative stress and exacerbated inflammatory responses. These changes may result in damage and degradation of articular cartilage, ultimately inducing OA.

Finally, we found that altered hemodynamics is also a significant indicator of chondrocyte activity. Recent statistical studies have demonstrated that hypertension and osteoarthritis are interrelated conditions (143). Articular cartilage lacks blood vessels and nerve endings; therefore, the nutrition necessary for its growth and maintenance primarily derives from the subchondral bone and synovium (144, 145). The synovium is richly vascularized and exhibits a high density of capillary distribution (146, 147). The metaphyseal trabecular bone within the subchondral region is also highly vascularized, containing a network of capillaries and sinuses (148, 149). Hypertension modifies the state of blood flow, leading to abnormal perfusion of these capillaries and sinuses, which in turn alters the hemodynamics of the subchondral bone and impairs the nutritional supply to the cartilage (150–152).

#### 2.2.2 Cartilage to cardiovascular

Chondrocytes can influence the heart and blood vessels by regulating specific cytokines. In healthy chondrocytes, the proliferation and differentiation of vascular smooth muscle cells are modulated through the Wnt/β-catenin signaling pathway. MiR-214-3p, a microRNA secreted by chondrocytes, plays a significant role in this process. Overexpression of miR-214-3p inhibits endothelial cell migration and angiogenesis, a process mediated through the regulation of the TrkB pathway. Activation of TrkB results in elevated levels of VEGF, which promotes endothelial cell migration and angiogenesis. In contrast, in osteoarthritic chondrocytes, the expression of miR-214-3p is down-regulated while TrkB levels are up-regulated, leading to increased VEGF secretion that facilitates endothelial cell migration and angiogenesis (97). Furthermore, in studies of cardiac fibrosis, miR-214-3p has been shown to prevent cardiac fibrosis by targeting NOD-like receptor family CARD domain containing 5 (NLRC5). It remains to be further investigated whether the risk of myocardial fibrosis is reduced in OA patients (98). Additionally, chondrocytes produce metabolites that influence the heart and blood vessels. The metabolic profile of chondrocytes in OA differs from that of normal chondrocytes, characterized by markedly increased glycolytic activity, which leads to elevated lactate production (99). This excess lactate enters the circulation, promoting angiogenesis by activating the PI3K/Akt pathway and subsequently upregulating VEGF expression (100).

The cardiovascular-cartilage axis represents a complex yet under-explored domain, with potential implications for both cardiovascular and cartilage health. The interactions within this axis are multifaceted, involving various factors such as metabolism, inflammation, and mechanical forces. Notably, shared influences, including angiogenic factors like VEGF, exhibit dual roles in cardiovascular repair and cartilage degradation. Thus, advancing strategies for tissue-specific modulation of angiogenesis, while balancing the requirements for cardiovascular and cartilage repair, is paramount for future research. Overcoming challenges related to target specificity, model limitations, and clinical translation bottlenecks is essential, with interdisciplinary collaboration and precision medicine technologies playing a crucial role.

### 2.3 Lung− cartilage axis

There exists a significant correlation between osteoarthritis and lung disease, as evidenced by numerous studies that highlight the interaction between lung and bone. Conditions such as lung cancer and pneumonia produce factors that influence cartilage cells (153, 154). Conversely, articular cartilage also generates factors that impact lung diseases. Therefore, the objective of this section is to elucidate the interactions between lung disease and articular cartilage.

#### 2.3.1 Lung to cartilage

Lung tumor cells can express lncRNA-MIR4435-2HG (50), which is associated with lymph node metastasis and enhances the metastatic capabilities of tumor cells (51). Additionally, this lncRNA promotes the proliferation of chondrocytes while inhibiting their apoptosis (50, 52). The COVID-19 pandemic represents the most significant global public health threat in the past five years (155), with infected individuals exhibiting markedly different primary conditions based on individual variability (156). Research indicates that patients with COVID-19 pneumonitis show elevated expression levels of the CXC chemokine receptor 4 (CXCR4) gene (157). CXCR4, a receptor located on the surface of cartilage (53), is activated by the binding of upstream inflammatory factors, which in turn activates the TGF-β/Smad2/3 signaling pathway and subsequently induces the release of matrix metalloproteinases, leading to cartilage degradation in osteoarthritis. Chondroitin sulfate (CS), an essential component of the ECM of cartilage, is closely linked to the metabolism of chondrocytes (158, 159).

#### 2.3.2 Cartilage to lung

Chondrocyte-derived factors and ECM components have been demonstrated to influence the development of lung diseases. The secreted glycoprotein YKL-40, produced by chondrocytes (101), is significantly elevated in the serum of patients with asthma and chronic obstructive pulmonary disease (COPD), where it serves as a crucial serum predictor for COPD (102). Additionally, YKL-40 is secreted by chondrocytes in osteoarthritic cartilage, which correlates with a higher prevalence of asthma and COPD (103). CS, an essential component of the cartilage extracellular matrix, is closely linked to chondrocyte metabolism (104). Evidence suggests that the higher the level of chondroitin sulfate in the lungs, the more severe the airway obstruction (105, 106). CS has been identified as playing a crucial role in certain severe lung diseases. Eleni et al. conducted an immunohistochemical analysis of lung tissues from patients who succumbed to COVID-19, revealing a significant elevation of chondroitin sulfate, particularly in the diffuse alveolar damage (DAD) region. Subsequent studies indicated that during instances of severe lung injury, the activity and expression of arylsulfatase B (ARSB), an enzyme essential for the degradation of chondroitin sulfate, were diminished (105). CS has been implicated in tissue remodeling across various diseases (160). Moreover, most current pharmacological treatments for OA aim to repair cartilage by promoting the regeneration of its ECM (161). However, these treatments may inadvertently lead to the non-specific deposition of CS in the lung interstitium, potentially increasing the risk of pulmonary fibrosis.

The interaction mechanism of the lung-cartilage axis involves inflammation, metabolism, and various other factors. Effective treatment must navigate the complexities of inter-organ regulation, model limitations, and potential drug side effects. The systemic effects of chronic inflammation and oxidative stress in the lungs are challenging to regulate locally; when targeted therapies are applied, they may inadvertently lead to dual lung-cartilage side effects. Lung hypoxia, as seen in conditions like COPD, results in elevated blood lactate and systemic lactate levels (162), which may activate chondrocyte MMP-13 expression by acidifying the joint microenvironment, potentially promoting cartilage degradation (163). Developing novel anti-inflammatory drugs that can simultaneously control lung inflammation and mitigate metabolite accumulation resulting from lung disease, without adversely impacting cartilage repair, presents a significant challenge.

### 2.4 Liver−cartilage axis

The liver is a crucial digestive organ in the human body, responsible for the production of various digestive enzymes and serving as a key hub for numerous physiological processes (164). However, the interactions between liver and cartilage metabolism remain underexplored and vary significantly under both physiological and pathological conditions. The following sections will summarize this organ axis.

#### 2.4.1 Liver to cartilage

Insulin-like growth factor-1 (IGF-1) is secreted by hepatocytes and plays a critical role in regulating the metabolism, proliferation, and growth of various cells in the body (70, 71). Studies have demonstrated its ability to influence the progression of OA by altering IGF-1 concentrations (72). IGF-1 inhibits NF-κB signaling by regulating the MAPK and PI3K/Akt signaling pathways in chondrocytes, thereby preventing chondrocyte apoptosis through the inhibition of ROS production, which in turn inhibits the progression of OA (73). Furthermore, non-alcoholic liver disease has been positively correlated with OA (165, 166), warranting further exploration of the mechanisms underlying the interaction between liver disease and cartilage. A long non-coding RNA, known as Long non-coding RNA highly up-regulated in liver cancer (lncRNA-HULC), is highly expressed in hepatocellular carcinoma. It sponges miR-101 by inhibiting the activation of the NF-κB and MAPK signaling pathways, ultimately protecting *in vitro* chondrocytes from TNF-α-induced inflammatory injury.

#### 2.4.2 Cartilage to liver

The mechanisms by which chondrocytes directly secrete cytokines to influence liver function *in vivo* remain inadequately explored. However, it is now well established that CS, which is produced from the degradation of the cartilage extracellular matrix in patients with OA, as well as certain drugs that promote the synthesis of the cartilage extracellular matrix, can adversely affect liver health. Notably, glucosamine and CS have been shown to exacerbate hepatocellular damage in individuals with chronic liver diseases (167) and may even obscure the risk associated with pre-existing hepatitis (168). This indicates that the use of chondrotherapeutic agents should be approached with caution in patients with cartilage damage and concurrent hepatitis, as it may hinder the efficacy of cartilage repair.

As a crucial metabolic organ, liver dysfunction can directly or indirectly harm cartilage through abnormalities in metabolites. Although several potential therapeutic targets have been identified, including IGF-1 and IL-6, which play significant roles in the liver-bone axis, the specific functions and efficacies of these targets in various liver diseases and conditions associated with the liver-cartilage axis require further investigation. Additionally, multiple compensatory mechanisms may influence the therapeutic outcomes. Therefore, identifying therapeutic targets that exhibit high specificity, effective efficacy, and minimal side effects remains a pressing challenge, necessitating more fundamental research and clinical trials to accurately screen and identify truly effective targets.

**Figure 2:**
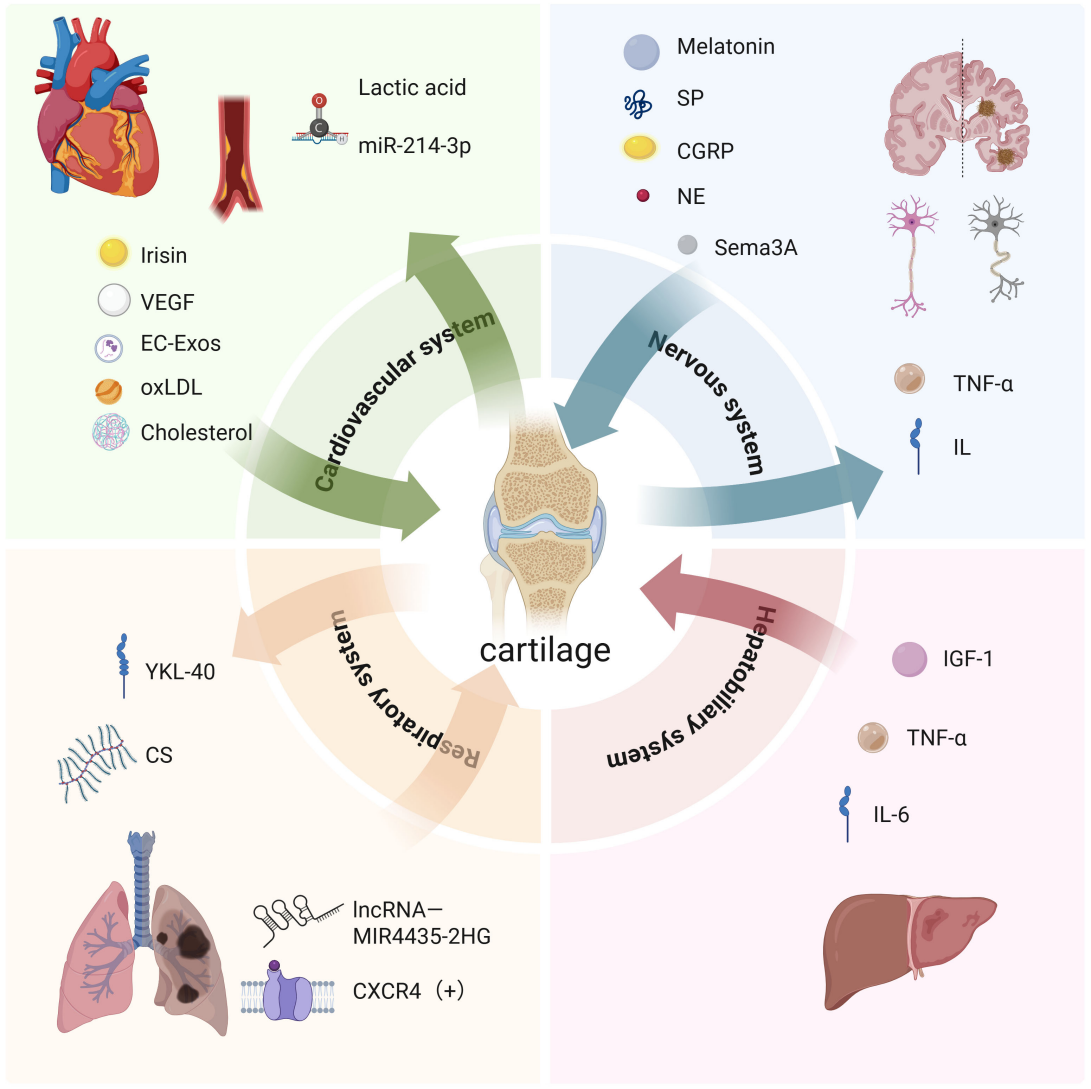
Overview of interaction between cartilage and brain, heart, lungs and liver.

The influence of other organs on cartilage is unidirectional, and no evidence has yet been found to suggest that cartilage interacts reciprocally with these organs. Nonetheless, we summarize this information for reference (**Figure 2**).

### 2.5 Intestine− cartilage axis

The intestinal tract serves multiple functions, including the digestion and absorption of food, as well as an endocrine role. It is also the body’s largest site of bacterial colonization. Intestinal peptides produced by the intestinal tract and microbial metabolites can significantly affect cartilage health. Maintaining a dynamic balance of intestinal flora is essential for cartilage health; any disruption of this balance can trigger various pathological processes throughout the body (169–173). Recent studies have indicated a significant impact of intestinal flora on the existence and progression of OA (174, 175). The gut microbiota(GM) and its metabolites can initiate systemic inflammation that impacts the joints through various pathways. For instance, bacterial components, such as lipopolysaccharides, can traverse the compromised intestinal barrier and enter the bloodstream, thereby activating the immune system and prompting the release of pro-inflammatory cytokines, including TNF-α, IL-1β, and IL-6. These cytokines accumulate locally in the joints, resulting in joint inflammation and cartilage degradation (176).

Glucagon-like peptide (GLP) is a hormone primarily produced by intestinal L-cells (74), classified as an enteroglucagon. *In vivo*, it activates the glucagon-like peptide-1 receptor (GLP-1R), leading to various downstream effects (75). Activation of GLP-1R has been shown to protect chondrocytes from endoplasmic reticulum stress and apoptosis induced by IL-1β or triglycerides (TGs). Furthermore, GLP inhibits apoptosis and endoplasmic reticulum stress via the PI3K/Akt signaling pathway. The activation of GLP-1R also suppresses the nuclear factor κB pathway in TG-treated chondrocytes, thereby reducing the release of inflammatory mediators such as IL-6 and tumor necrosis factor α, and decreasing matrix catabolism (76, 77). Recent studies have elucidated the mechanism by which the activation of GLP-1R alleviates osteoarthritis (OA), providing evidence that farnesoid X receptor (FXR) serves as a viable therapeutic target for this purpose through the enhancement of GLP-1 levels. Further research has identified ursodeoxycholic acid (UDCA) as a drug that targets FXR activation, which subsequently promotes intestinal production of GLP-1 and activates GLP-1R, thereby facilitating the repair of chondrocytes (78).

The impact of the GM on the systems and tissues of the human body is profound. GM influences cartilage health through multifaceted mechanisms, including metabolites, immune regulation, barrier maintenance, and neuroendocrine interactions. We summarize the effects of several factors—namely, short-chain fatty acids(SCFAs), capsiates(CAT), tryptophan, and extracellular vesicles (EVs)—on cartilage production. The gut microbiota produces SCFAs, which have been demonstrated to modulate inflammation and may confer protective effects on chondrocytes. Notably, levels of total fatty acids, saturated fatty acids, and linoleic acid entering the gut have been significantly associated with a reduced risk of osteoarthritis. Following their breakdown and absorption, short-chain fatty acids, among others, have been found to exert significant anti-osteoarthritis effects (177, 178). SCFAs can act directly on chondrocytes after entering the bloodstream or indirectly protect against chondrocyte apoptosis and matrix degradation by inhibiting osteoclast activity and modulating the inflammatory response, thereby enhancing cartilage repair (79, 80). CAT, another metabolite produced by intestinal flora, inhibit HIF-1α expression and reduce iron-death-dependent chondrocyte damage through the activation of Solute Carrier Family 2 Member 1 (SLC2A1) (81). Intestinal tryptophan metabolites, such as indole-3-acetic acid (IAA) and indole-3-pyruvate (IPA), function as aryl hydrocarbon receptor (AhR) ligands, activating the AhR and subsequently initiating the Wnt/β-catenin signaling pathway, which significantly promotes chondrocyte proliferation (82).

GM serves as a double-edged sword; when in a balanced state, it protects joint health. Conversely, when the gut flora becomes imbalanced, various harmful substances or factors can enter the bloodstream, ultimately affecting the joints and promoting the formation of OA. While some studies have indicated that probiotics and prebiotics may confer benefits for arthritis, there remains a lack of uniform standards and consensus regarding the most effective probiotic strains, their combinations, dosages, and duration of use. Fecal transplants represent an emerging strategy for modulating gut microbiota; however, their application in the treatment of arthritis is still in the exploratory phase.

### 2.6 Kidney-cartilage axis

The kidney is the primary organ responsible for regulating calcium and phosphorus metabolism in the body, thereby influencing calcium and phosphorus homeostasis, which in turn affects bone mineral deposition and the quality of bone. Although there are fewer studies examining the effects of the kidney and chronic kidney disease (CKD) on cartilage, fibroblast growth factor 23 (FGF23) has emerged as a significant factor linking bone metabolism with renal calcium and phosphorus metabolism. FGF23, a hormone predominantly secreted by osteoblasts (107), regulates blood phosphate levels by promoting renal phosphate excretion and inhibiting the production of 1,25-dihydroxyvitamin D3 [1,25(OH)2D] (179, 180). It achieves this by suppressing the expression of renal phosphate transporter proteins in the proximal renal tubules, thus maintaining the balance between bone synthesis and catabolism. Recent studies have increasingly illuminated the relationship between FGF23 and arthritis, with evidence suggesting that FGF23 enhances the expression of ColX, MMP13, and MMP9 in chondrocytes through the modulation of the Wnt/β-catenin signaling pathway, ultimately contributing to OA (181, 182). Furthermore, it has been demonstrated that FGF23 plays a role in preserving the chondrocyte phenotype in OA (182). Here, we propose the hypothesis that the kidney influences FGF23 expression by modulating calcium and phosphorus metabolism; however, the validity of this hypothesis and the underlying mechanisms merit further investigation.

### 2.7 Gonad-cartilage axis

Sex hormones, particularly estrogen, play a crucial role in the development and progression of OA (183, 184). As research on OA continues to advance, the gender differences in prevalence and symptom burden are increasingly concerning, particularly given that postmenopausal women exhibit a higher prevalence of OA. Subsequent studies have demonstrated that estrogen has a protective effect on chondrocytes (185). Animal studies indicate that estrogen supplementation significantly reduces the expression of matrix metalloproteinase 13 (MMP13) and decreases chondrocyte apoptosis in female mice (186). *In vitro* studies have demonstrated that estrogen activates AMP-activated protein kinase (AMPK) and inhibits the phosphorylation of mammalian target of rapamycin (mTOR) by enhancing the expression of SIRT1 in chondrocytes. This process subsequently activates AMPK and suppresses mTOR phosphorylation, leading to a signaling cascade that promotes mitochondrial autophagy in chondrocytes. Consequently, this cascade enhances chondrocyte proliferation and contributes to the improvement of OA (187). *In vivo*, Estrogen exerts its effects by binding to estrogen receptors (ER) present in the tissues surrounding the joints, specifically binding to estrogen receptor beta (ERβ), which negatively regulates arthritis progression by inhibiting the activation of the NF-κB pathway in synoviocytes (188). Recent studies have revealed a mutual antagonism between estrogen and IL-1β, with IL-1β down-regulating the quantity and functionality of ERα in chondrocytes. The activation of ERα by estradiol (E2) or selective estrogen receptor modulators (SERMs) induces anti-inflammatory effects in these cells, ultimately inhibiting nitric oxide (NO) production and the expression of inducible nitric oxide synthase (iNOS), thereby mitigating chondrocyte damage (83). Previously, it was believed that estrogen could influence chondrocyte metabolism solely through ER (84, 85). However, recent findings have demonstrated that estrogen can also exert chondroprotective effects by activating the G protein-coupled estrogen receptor (GPER). On one hand, by inhibiting the mechanical stress-mediated activation of Piezo1 channels, estrogen reduces actin polymerization, thus inhibiting chondrocyte apoptosis, which suggests a protective role for estrogen in post-traumatic arthritis (84, 85). On the other hand, the activation of GPER decreases intracellular levels of ROS and lipid peroxides, which in turn alleviates intracellular iron overload in chondrocytes, thereby providing additional protection to these cells (86).

### 2.8 Pancreas−cartilage axis

**Figure 3:**
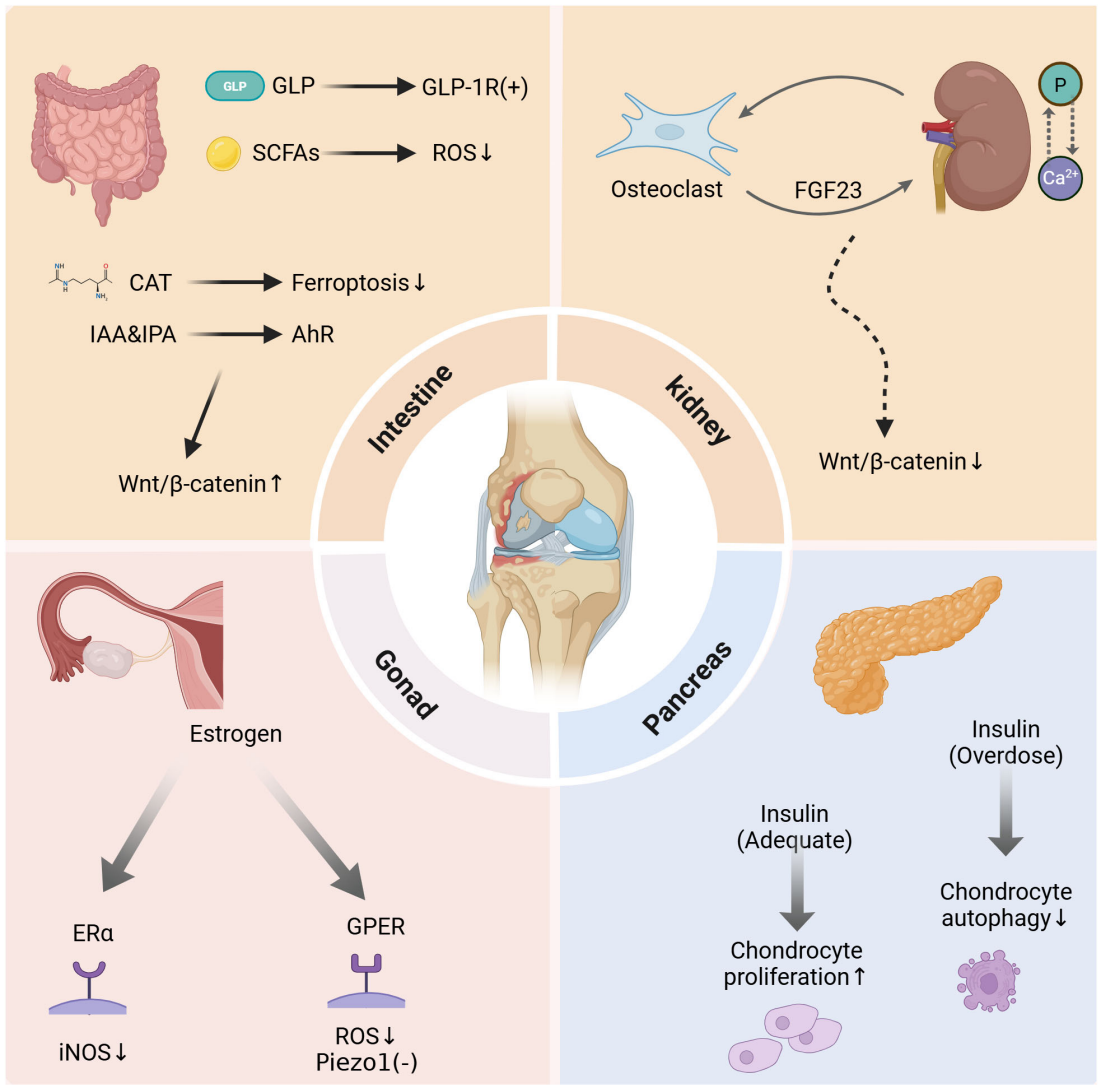
Overview of Intestinal, renal, gonadal, pancreatic, adipose, muscular, and skeletal effects on cartilage.

The pancreas serves as a crucial digestive organ and a regulator of blood glucose levels in the body. Insulin, one of the most significant hormones for chondrocytes, plays a pivotal role in this context. Previous research has established a link between insulin and OA, highlighting that conditions such as diabetes and metabolic syndrome alter the secretion of insulin and glucagon. These alterations can lead to changes in body weight, where weight gain exacerbates weight-bearing stress on the hip and knee joints, potentially resulting in cartilage damage and accelerating the development of OA (189–193). Recent studies have revealed that insulin influences the metabolism of chondrocytes and synovial cells in the joints through various mechanisms, thereby impacting joint health. In recent years, it has been discovered that insulin influences the metabolism of chondrocytes and synovial cells in the joints in various ways, thereby impacting joint health. Researchers have identified insulin-associated receptors on chondrocytes and synovial cells (87–89). An increasing number of studies have demonstrated that the effects of insulin on chondrocytes are complex and biphasic (90). Some studies indicate that moderate levels of insulin exhibit anti-apoptotic effects on chondrocytes and promote their proliferation in animal models (88, 91). However, high concentrations of insulin may down-regulate cartilage proliferation and repair by inhibiting chondrocyte autophagy (92). Additionally, insulin can exacerbate the progression of OA by influencing fibroblast-like synoviocytes (FLS), leading to increased secretion of pro-inflammatory cytokines (93). Interestingly, insulin appears to selectively protect hand joints by inhibiting the release of catabolic enzymes from FLS into the synovial fluid; however, it remains to be determined whether this effect extends to systemic joints (94) (**Figure 3**).

### 2.9 Adipose tissue-cartilage axis

Adiposity is closely linked to the progression of OA. A high body fat percentage in obese patients constitutes a significant risk factor for the development of OA, as the increased pressure on the knee joint exacerbates the wear and tear on articular cartilage. Furthermore, adipose tissue not only influences chondrocyte metabolism but has also been implicated in the progression of OA through the action of adipokines (194). Adipose tissue regulates chondrocyte metabolism via the secretion of free fatty acids (FFA) (195, 196), lipocalin (197–199), and leptin (200). Notably, lipocalin has been shown to protect chondrocytes from hydrogen peroxide (H2O2)-induced apoptosis by activating autophagy through the AMPK/mTOR signaling pathway (111). Additionally, infrapatellar fat pad (IPFP) mesenchymal stromal cell-derived exosomes (MSCIPFP-Exos) transport miR-100-5p into chondrocytes, thereby inhibiting apoptosis by suppressing the mTOR signaling pathway (117). FFA has been found to cause chondrocyte damage by upregulating the expression of nuclear protein 1 (Nupr1) on the surface of chondrocytes (112). In an *in vitro* assay, an investigator discovered that leptin promotes chondrocyte proliferation and gene expression in a dose-dependent manner, with maximal effects observed at a leptin concentration of 100 ng/mL. Leptin is detectable in the synovial fluid of patients with OA (113); however, contrary to *in vitro* findings, its levels in synovial fluid are positively correlated with the severity of OA (114). Additionally, leptin has been shown to promote the catabolism of articular cartilage and functions as a pro-inflammatory factor within this tissue (115). It mediates the production of NLRP3 inflammatory vesicles in chondrocytes through reactive ROS production, ultimately leading to the upregulation of various inflammatory factors (e.g., IL-1β, IL-6, IL-8) and resulting in cartilage degradation and joint inflammation (116).

### 2.10 Muscle−cartilage axis

The relationship between muscle strength and OA has garnered considerable attention in recent years (201). Exercise therapy has long been recognized as an effective treatment for musculoskeletal disorders. It is suggested that aerobic exercise reduces the accumulation of lipid peroxides in the body (202). Furthermore, numerous studies have demonstrated that stronger lower extremity muscles protect the knee joint, thereby inhibiting the progression of OA (203). However, for patients who have already developed OA, pain often limits their movement and exercise, leading to further muscle weakening and exacerbating the progression of the disease (204–207). An increasing body of research indicates that the functional state of muscle can significantly influence skeletal metabolism (208). Folliculin interacting protein 1 (FNIP1) has been identified as a crucial regulator of skeletal muscle activity and bone metabolism within muscle tissue (118, 119). Additionally, skeletal muscle mesenchymal progenitors (MPs) play a vital role in the regenerative processes of skeletal muscle (209). It has been observed that muscle MP cells can exuberantly produce ECM under specific conditions (210), representing a promising therapeutic avenue for cartilage damage repair.

### 2.11 Bone−cartilage axis

The structure adjacent to the articular cartilage is the subchondral bone. Healthy cartilage is spatially interconnected with its subchondral bone, and studies have demonstrated a biosignaling relationship between the two (211). Subchondral bone primarily consists of cancellous bone and adjacent bone marrow, where osteoblasts and osteoclasts exist in a dynamic equilibrium. When this equilibrium is disrupted, it can induce apoptosis of chondrocytes and accelerate the progression of OA (212). Osteoblasts in physiological subchondral bone enhance glycolytic metabolism in chondrocytes via the MAPK/HIF-1 pathway (213). However, transforming growth factor β (TGF-β) secreted in subchondral bone serves as a crucial regulator (120); abnormal elevation of TGF-β accelerates cartilage degeneration and subchondral bone resorption (121). Similarly, osteoclasts, under physiological conditions, exert a mutual protective effect with chondrocytes (214). Yet, when osteoclasts interact with abnormal cartilage, they degrade the cartilage matrix by secreting MMP8 and MMP9, further exacerbating cartilage degeneration in osteoarthritis and other diseases (215). It is evident that the health of the subchondral bone directly influences the health of the cartilage. The relationship between the two can be likened to that of soil and crops; the health of the former is conducive to the healthy proliferation of cartilage in terms of both spatial and mechanistic support. Furthermore, the dynamics of osteoblasts and osteoclasts in the bone marrow represent a promising area for future research.

## 3 Strategies for restoring cartilage by intervening in extraosseous organs

Research on cartilage repair by modulating the functional status of extraosseous organs is gaining traction. An analysis of the aforementioned studies indicates that chondrocytes are significantly involved in crosstalk with the brain, heart, lungs, and other systems. Interventions aimed at promoting chondrocyte growth, inhibiting apoptosis, and enhancing the production of extracellular matrix in cartilage through the modulation of organ-specific or highly expressed factors may be pivotal in preventing OA.

Transorganic cytokines exhibiting chondroprotective effects and the mechanisms by which they facilitate chondrocyte proliferation have been elucidated. Sema3A, secreted by sensory neurons, serves to protect chondrocytes and maintains chondrocyte homeostasis through the activation of the PI3K pathway (69). Nerve growth factor (NGF) is a significant mediator of pain in OA, and its antibodies have been extensively validated as a therapeutic approach for pain relief (216). Recent research has achieved a breakthrough in the regulation of biological circadian rhythms; bone morphogenetic protein 2 (BMP2) and runt-related transcription factor 2 (RUNX2) are key initiators of osteocalcin transcription, and their expression levels fluctuate with photoperiod and melatonin, thereby promoting chondrocyte repair (217). MT protects cells from pathological death by scavenging reactive oxygen species in tissues and promoting the expression of glutathione and antioxidant enzymes (92). Intracellularly, MT inhibits the release of TNFα and interleukin-1 beta (IL-1β) in the joints (218), while simultaneously promoting the production of the ECM by activating the TGF-β signaling pathway (219). The mechanism of action of irisin involves enhancing mitochondrial function in chondrocytes. Research has demonstrated that appropriate exercise stimulates the synthesis and release of irisin *in vivo*. The specific repair mechanism of irisin on chondrocytes is linked to the reduction of the expression of the chondrocyte senescence marker P21 through the inhibition of the PI3K/Akt signaling pathway, thereby alleviating chondrocyte degeneration (41, 220). lncRNAs and microRNAs, which are typically produced by non-chondrocytes and often associated with tumor cells, have been shown to increase tumor aggressiveness. However, extracting these RNAs and transferring them to chondrocytes promotes chondrocyte proliferation. Collectively, these cytokines exhibit chondrocyte-protective effects. Future research will focus on exploring their modes of delivery, synthesizing stable compounds, and constructing suitable drug delivery systems, which is of significant interest.

Cellular receptors in their non-free state, along with the downstream effects triggered by their activation, can rejuvenate chondrocytes without isolating them from the body. The activation of α-1-adrenergic receptors is known to promote chondrocyte apoptosis, while α-adrenergic receptor (α-AR) antagonists, particularly phentolamine, have been shown to stimulate chondrogenesis and induce hypertrophy (221). Prostaglandin E2 (PGE2), which regulates vasodilation and permeability, and Signal Transducer and Activator of Transcription 3 (STAT3), secreted by vascular endothelial cells, both promote H-type angiogenesis in the subchondral bone, exacerbating OA (222, 223). Recent studies indicate that the mechanosensitive receptor Piezo1 plays a pivotal role in both angiogenesis and cartilage homeostasis (223). Specifically, Piezo1 promotes the redistribution of blood flow and increases microvessel density in muscle, while sensing changes in mechanical stress on the surface of chondrocytes, which in turn activates downstream signaling pathways altered by Ca2+ influx, thereby promoting chondrocyte apoptosis (224, 225). The activation of the GPER has been shown to inhibit the mechanical stress-mediated RhoA/LIMK/cofilin pathway, ultimately reducing the expression of the Piezo1 protein and alleviating apoptosis in chondrocytes (84). β-Hydroxybutyrate (β-HB), a readily available energy carrier produced by adipocytes, enhances chondrocyte autophagy and diminishes chondrocyte apoptosis by activating the Erb-B2 receptor tyrosine kinase 3 (ERBB3) signaling pathway. This action ameliorates OA without leading to hepatic pathology. Furthermore, liraglutide has been demonstrated to activate downstream pathways upon interaction with the GLP-1R, subsequently inducing chondrogenesis and reducing chondrocyte apoptosis. GLP-1 agonists also contribute to the improvement of sarcopenia and the increase of bone density in the elderly, indirectly enhancing and preventing the progression of OA (226). It is important to note that these receptors are not exclusively expressed by chondrocytes; different receptor proteins exhibit distinct activation effects in various cell types. Consequently, the investigation of drugs targeting these chondrocyte receptors remains a significant focus within pharmacological research.

Optimizing the structure of intestinal flora and enhancing the autonomous absorption of nutrients conducive to cartilage repair through improvements in dietary composition is a treatment approach that requires time to manifest its effects. Supplementing the daily diet with vitamin B1, which is absorbed into the bloodstream via the intestinal tract, can significantly alleviate the symptoms of OA by modulating macrophage polarization and thereby inhibiting chondrocyte apoptosis. Furthermore, promoting the production of SCFAs in the intestinal tract is crucial; dietary fiber serves as the primary substrate for the fermentative production of SCFAs by intestinal flora. A high-fiber diet, defined as an intake of more than 30 grams of fiber per day (227), can substantially increase the concentration of SCFAs in feces. Additionally, the intake of probiotics (e.g., Lactobacillus, Bifidobacterium) can synergistically enhance these fermentation effects, resulting in increased SCFA production and inhibiting the proliferation of harmful bacteria (228, 229). This interplay can effectively promote cartilage repair through the action of SCFAs in the bloodstream.

Bone-cartilage grafting represents a promising approach for the repair of arthropathic lesions, integrating both mechanical and molecular biological strategies. This technique utilizes healthy cartilage and subchondral bone tissues, wherein healthy cartilage-bone plugs are transplanted into the injured area. These transplanted bone plugs not only provide initial mechanical support and reduce joint stress concentration but also supply active chondrocytes and bone matrix, thereby promoting chondrocyte migration and matrix deposition in the affected region. In OA, the balance between osteoblasts and osteoclasts in cartilage and subchondral bone is disrupted; however, the transplantation of bone-cartilage grafts from healthy areas can enhance the subchondral microenvironment and facilitate the formation of peripheral cartilage. Future research is focused on improving graft survival rates through gene editing techniques, such as CRISPR technology, which can knock down IL-1 receptor expression in chondrocytes to enhance their anti-inflammatory capabilities. Moreover, the potential discovery of new loci associated with pathological chondrocyte death underscores the importance of gene editing in this field.

Exosomes derived from various sources present a universal therapeutic solution. As previously discussed, numerous organs and tissues can ameliorate OA by enhancing chondrocyte viability and promoting their proliferation through characteristic mesenchymal stem cell (MSC)-derived exosomes. Additionally, exosomes exhibit extremely low immunogenicity, which renders allogeneic interspecies therapy feasible and underscores their significant therapeutic potential. Future advancements in the efficient purification of exosomes from diverse sources, as well as in the engineering and targeting of these exosomes, promise to enhance their therapeutic efficacy.

Future studies should investigate the mechanisms underlying the effects of various organs on cartilage. Numerous pathways remain to be explored to enhance these therapeutic approaches and to develop precise therapeutic strategies aimed at optimizing clinical efficacy. Such advancements will make the prevention of OA a more attainable goal in the future.

The inter-crosstalk between multiple organs constitutes a more complex system that is critical to the health impact of the joints. The brain-gut-bone-joint axis has been extensively studied, with numerous studies confirming its close relationship with neurodegenerative diseases. Dysbiosis of the intestinal microbiota can lead to abnormalities in metabolites, such as SCFAs and lipopolysaccharides (LPS), which may affect the synthesis and release of neurotransmitters, resulting in neuroinflammation and neurodegeneration (230). Furthermore, dysbiosis or the accumulation of undesirable metabolites can contribute to the degeneration of articular cartilage. The brain-gut-bone-joint axis encompasses complex interactions among multiple systems, with significant variations in genes, composition of intestinal microbiota, lifestyles, dietary habits, and other factors among individuals. These variations complicate the exploration of this axis, although the potential for future research in this area is promising. Additionally, multifactorial interactions with other organs warrant further investigation.

## 4 Conclusions and future perspectives

Contrary to traditional perceptions of OA, recent studies characterize OA more as a metabolic syndrome disease (231). In this context, various extraosseous organs throughout the body influence chondrocyte value addition and apoptosis through multiple cytokines, EVs, hormones, and metabolites. Therefore, assessing the functional status of different organ systems is crucial when focusing on OA.

Numerous potential therapeutic approaches and the potential interactions between various systemic diseases have emerged from the perspective of the organ-cartilage axis. The administration of chondroitin sulfate should be individualized for OA patients with concurrent pulmonary fibrotic disease, as sensory neurons not only play a role in the generation of nociceptive sensations but also enhance the viability of chondrocytes.

Exosomes derived from various organ sources represent promising therapeutic tools, yet our understanding of them remains limited. Most exosomes enter chondrocytes by delivering miRNAs and proteins that promote cellular proliferation. In future research, we will selectively utilize exosomes from specific sources based on the distinct damage mechanisms affecting chondrocytes. However, it is important to note that not all exosomes facilitate chondrocyte proliferation or inhibit apoptosis; for instance, EC-Exos can induce chondrocyte apoptosis. Furthermore, exosomes derived from probiotic sources exhibit significant therapeutic potential for human diseases, presenting a promising avenue for investigating the influence of gut flora on human health.

OA involves lesions beyond mere cartilage damage, including synovial inflammation, bony changes in the subchondral bone, ligamentous damage, and degeneration of the surrounding muscles. There remains a significant research gap regarding the connection between pathological changes in these tissues and the organ axis. Comprehensive exploration of the mechanisms underlying these complex interactions is an essential task in biomedical research.

This review proposes that OA should be perceived as a systemic disease, where its progression reflects the functional state of all organs in the body. Future treatment strategies and research on OA may extend beyond bone metabolism, cartilage repair, and synovial regeneration. Instead, multidisciplinary and cross-disciplinary exploration, along with mechanistic research, is likely to become a mainstream approach.

## Glossary

1,25(OH)2D: 1,25-dihydroxyvitamin D3
AD: Alzheimer’s disease
ADRD: AD-related dementia
AhR: Aryl hydrocarbon receptor
AR: Androgen receptor
ARC KISS1: KISS1 neurons in the arcuate nucleus
Aβ: Amyloid beta
BBB: Blood-brain barrier
BMD: Bone mineral density
BMP2: Bone morphogenetic protein 2
CAT: Capsiates
CCN3: Cellular communication network factor 3
CGRP: Calcitonin gene-related peptide
CKD: Chronic kidney disease
COPD: Chronic obstructive pulmonary disease
CS: Chondroitin sulfate
CVD: Cardiovascular disease
CXCR4: CXC chemokine receptor 4
E2: Estradiol
EC-Exos: Exosomes derived from vascular endothelial cells
ECM: Extracellular matrix
ER: Estrogen receptor
ERBB3: Erb-B2 receptor tyrosine kinase 3
ERβ: Estrogen receptor beta
EVs: Extracellular vesicles
FFA: Free fatty acids
FGF23: Fibroblast growth factor 23
FLS: Fibroblast-like synoviocytes
FNDC5: Fibronectin type III domain-containing protein 5
FNIP1: Folliculin interacting protein 1
GLP: Glucagon-like peptide
GLP-1R: Glucagon-like peptide-1 receptor
GM: Gut microbiota
GPER: G protein-coupled estrogen receptor
H2O2: Hydrogen peroxide
IAA: Indole-3-acetic acid
IGF-1: Insulin-like growth factor-1
IL-1β: Interleukin-1 beta
iNOS: Inducible nitric oxide synthase
IPA: Indole-3-pyruvate
IPFP: Infrapatellar fat pad
KOA: Knee osteoarthritis
LDL: Low-density lipoprotein
LPS: Lipopolysaccharides
lncRNA-HULC: Long non-coding RNA highly up-regulated in liver cancer
LRP3: Low-density lipoprotein receptor-related protein 3
MCP-1: Monocyte chemoattractant protein 1
MMP13: Matrix metalloproteinase 13
MPs: Mesenchymal progenitors
MSC: Mesenchymal stem cell
MSCIPFP-Exos: Infrapatellar fat pad mesenchymal stromal cell-derived exosomes
MT: Melatonin
NFTs: Neurofibrillary tangles
NGF: Nerve Growth Factor
NLRC5: NOD-like receptor family CARD domain containing 5
NO: Nitric oxide
Nupr1: Nuclear protein 1
OA: Osteoarthritis
oxLDL: Oxidized LDL
PGE2: Prostaglandin E2
PTH: Parathyroid hormone
RORα: Retinoic acid-related orphan receptor α
ROS: Reactive oxygen species
RUNX2: Runt-related transcription factor 2
SCFAs: Short-chain fatty acids
Sema3A: Semaphorin 3A
SERMs: Selective estrogen receptor modulators
SLC2A1: Solute Carrier Family 2 Member 1
SP: Substance P
STAT3: Signal Transducer and Activator of Transcription 3
TFEB: Transcription factor EB
TGF-β: Transforming growth factor β
TGs: Triglycerides
TNF-α: Tumor necrosis factor-alpha
VEGF: Vascular endothelial growth factor
α-AR: α-Adrenergic receptor
β-HB: β-Hydroxybutyrate.
